# Cardiovascular Disease and Mental Distress Among Ethnic Groups in Kyrgyzstan

**DOI:** 10.3389/fpubh.2021.489092

**Published:** 2021-05-04

**Authors:** Hossain Syed Azfar, Kenesh O. Dzhusupov, Hans Orru, Steven Nordin, Maria Nordin, Kati Orru

**Affiliations:** ^1^Department of Family Medicine, International School of Medicine, Bishkek, Kyrgyzstan; ^2^Department of Public Health, International School of Medicine, Bishkek, Kyrgyzstan; ^3^Department of Public Health and Clinical Medicine, Umeå University, Umeå, Sweden; ^4^Institute of Family Medicine and Public Health, University of Tartu, Tartu, Estonia; ^5^Department of Psychology, Umeå University, Umeå, Sweden; ^6^Institute of Social Studies, University of Tartu, Tartu, Estonia

**Keywords:** mental distress, cardiovascular disease, ethnicity, Central Asia, minority

## Abstract

The purpose of this study was to characterize different ethnic groups in Kyrgyzstan regarding cardiovascular disease (CVD) and mental distress, and to investigate the association between CVD and mental distress. The mental distress was measured in terms of sleep disturbance, burnout, and stress.

**Materials and Methods:** A cross-sectional study was carried out among six ethnic groups in Kyrgyzstan, aged 18 years and above. The sample was stratified for age, education, family status, and income. We used the Karolinska Sleep Questionnaire to assess sleep disturbance, the physical and emotional subscale of the Shirom Melamed Burnout Questionnaire to assess burnout, and the 10-item Perceived Stress Scale to assess perceived stress.

**Results:** The distribution of CVD differed significantly between the six ethnic groups, with higher prevalence among East Europeans, and Western Asians and lower among Other minorities and Central Asians. In all ethnic groups in Kyrgyzstan, individuals with CVD had increased odds of sleep disturbance and burnout. There was a significant difference in burnout and stress between persons with and without CVD in Kyrgyz and East European ethnic groups.

**Conclusion:** There was a significant difference in burnout and stress between persons with and without CVD in Kyrgyz and East European ethnic groups. In addition to CVD prevention, mitigating sleep disturbance and preventing burnout in the general population should be aimed at in public health measures.

## Introduction

Cardiovascular diseases (CVDs) are the worldwide leading cause of mortality (1, 2). Globally, the highest CVD mortality rates are found in Ukraine, Russia, and Central-Asia (63, 55, 63–42% in 2016, respectively) (3). Those post-Soviet countries like Kyrgyzstan [with a population of 6.3 million in 2018 (4)] have experienced volatile economic and political transitions that make up challenging socioeconomic conditions for health and well-being in the multi-ethnic population.

The main risk factors for CVD include physical inactivity, obesity, unhealthy diet, smoking, drug abuse, hypertension, and lipid abnormalities (5–11). Prior studies have shown lower CVD mortality attributable to dietary risk in Kyrgyzstan and among Central Asians (Kazakhs, Tajiks, and Kyrgyzs) compared to East Europeans (Russians, Ukranians, and Belorussians) and Western Asians (Georgians, Azerbaijani) (12). There are also gender differences as women have lower incidence levels and develop the disease later than men (13, 14). Their protective mechanisms against CVD are mainly associated with sex hormone (e.g., estrogen) levels as the incidence and severity of CVD increase in women during post-menopause period (15). At the same time, gender behavioral differences may play an important role. As women visit physicians more often, their diseases are registered more frequently, whereas men may not visit a physician until it may be too late (16).

Apart from those, various social factors may contribute to differences in CVD incidence. Individuals with low socioeconomic status experience higher rates of CVD burden and mortality (17–21). Low level of educational attainment is associated with high prevalence of cardiovascular risk factors, high CVD incidence, and CVD mortality (19, 22). In Kyrgyzstan, in 2017, the Gini coefficient, that is a measure of inequality among levels of income, was 26.8 (23). This reflects relatively good equality in income distribution since the country residents have a similar relatively low standard of living.

It has been indicated that socioeconomic inequalities are more prevalent among minority ethnic populations who have high prevalence of CVD and related risk factors (24), and this inequality is growing globally (25–28). Ethnic minorities also experience more barriers to access a CVD diagnosis (29), poorer recording of clinical data (30), receive lower quality of health care, and have poorer health outcomes (31). Carson and colleagues showed that ethnicity is an important predictor of hypertension (32). Kontsevaya et al. found that among Kyrgyz women, arterial hypertension prevalence was significantly lower than in their Russian peers (36.8 vs. 46.2%, respectively) (33). At the same time, among Kazakh and Kyrgyz men, systolic blood pressure was significantly higher than in Russians (33).

It is well-documented that social factors may moderate stress level (34). Chronic psychological stress is associated with a greater risk of depression, autoimmune diseases, respiratory infections as well as coronary heart disease (35, 36). Stress as a complaint is related to anxiety (37) and depression (38). Psychosocial factors such as depression and low social support are in turn established risk factors for heart disease (36, 39) and have been associated with high risk of adverse cardiovascular outcomes (35, 40–44), and mortality among patients with CVD (45, 46). Mental distress is associated to social factors (poverty and unemployment), ethnicity, gender, age, and disability (47, 48).

Stress may perpetuate sleep disturbance, as complaints in burnout (49) and symptoms of insomnia (50), may lead to poor sleep, worry, and increase of blood pressure (51, 52). Whereas, stress fluctuates strongly since it is a necessity in daily life in coping with everyday hassles, chronic stress that results in burnout may be a particular risk factor for chronic diseases (53, 54). Sleep disturbance is both an initiator and a consequence of burnout and depression (51, 55–57). As the result of long-term inflammatory processes due to mental distress, plaque formation on the vascular walls in atherosclerosis may eventually lead to a CVD event (58–60). The fact that stress also underlies burnout and sleep disturbance, highlights the importance of these conditions on persons with CVD.

The current study aimed at characterizing different ethnic groups in Kyrgyzstan with respect to CVD and the mental distress conditions including sleep disturbance, burnout, and stress, and to investigate the association between CVD and mental distress. Based on earlier evidence, we infer that some ethnic groups are more likely than other to contract CVD. The present study tested the hypotheses of the minorities, compared to dominant Kyrgyz ethnic group, being more likely to suffer from CVD and/or having higher levels of mental distress. Following Mezick et al. (61), Grandner et al. (62), Slopen et al. (63), and Johnson et al. (64), we expected ethnic-group differences also in sleep disturbance.

## Materials and Methods

### Data Collection

A sample of 694 individuals aged 18 years and older visiting polyclinics (Centers of Family Medicine) and health care centers were invited to participate in a study entitled “Health status of ethnic minorities in Kyrgyzstan.” We chose five polyclinics in the suburban areas, where representatives of minorities mainly reside in Bishkek. Kyrgyzs as a control group were recruited from the same facilities. We used a questionnaire with 47 questions to explore the health status, behavioral and psychological determinants and prevalence of CVD, body mass index (BMI), age, gender, education level, ethnicity, and income. Informed consent was obtained from participants after explaining the study aims, voluntariness of participation, and anonymised data processing. The respondents answered the questionnaire and could ask for assistance or explanations from the study leader.

The initial sample included 1,200 participants. With a response rate of 57.8%, this resulted in 694 respondents. We used random sampling stratified for ethnicity, age, education, and gender. Ethnicity was asked as open question: “Please, indicate your ethnicity:…” [see also Phinney and Ong (65)]. In Kyrgyzstan, the Kyrgyz comprise 73.3% of the population. Other major ethnic groups include Russians (5.6%) concentrated in the north, and Uzbeks (14.7%) living in the south. Small, but noticeable minorities include Dungans (1.1%), Uyghurs (0.9%), Tajiks (0.9%), Kazakhs (0.6%), and Koreans (0.3%). Other small ethnic minorities make up 2.6% of the population (4). Following this, the participants were divided into six groups based on their ethnicity. Kyrgyz people, (1) “Kyrgyz,” functioned as a control group for comparison with the other ethnic groups. Due to their similarity in religious background and geographical origins we grouped five additional ethnic groups as follows: (2) “East Europeans”: Russian, Byelorussian, and Ukrainian; (3) “Central Asians”: Uzbek, Kazakh, Tatar; (4) “East Asians”: Korean; (5) “Western Asians”: Georgian, Armenian, Turk, and Azerbaijan; and (6) “Other minorities”: Dungan and Uyghur. In the initial sample of 1,200 participants, we aimed at ethnic group proportions of 40% Kyrgyz, 20% East Europeans, 20% Central Asians, 5% East Asians, 5% Western Asians, and 10% Other minorities. The final sample of respondents consisted of 31.3% Kyrgyz, 34.4% East Europeans, 16.4% Central Asians, 5.5% East Asians, 3.3% Western Asians, and 9.1% Other minorities. Regarding age groups, we aimed at equal proportions of age groups, resulting in 24.9% aged 18-29, 24.4% aged 30-39, 17.3% aged 40-49, 17.3% aged 50-59, and 16.1% aged ≥ 60 years. Regarding education, we aimed at the proportions 26% with higher education, 68% with high school education, and 5% with elementary or secondary school. In the final sample, higher education was overrepresented, 41%, and the proportions were 57.2% with high school education, and 1.6% with elementary education. In the final sample male sex are slightly under-represented (43.5%).

The 694 respondents were distributed across the ethnic groups: Kyrgyzs (control, 217 individuals, mean age = 39.2 ± 14.8), East Europeans (239 individuals, mean age = 48.5 ± 15.7), East Asians (38 individuals, mean age = 43.4 ± 20.6), Central Asians (114 individuals, mean age = 38.1 ± 14.7), Western Asians (23 individuals, mean age = 43.2 ± 13.3), and Other Minorities (63 individuals, mean age = 34.3 ± 14.3).

### Questionnaire Instruments

The questionnaire used was in the Russian language. Socio-demographic variables were assessed following the Guidelines for Handling the Harmonized Questionnaire (66), and anthropometric data (e.g., height and weight) were assessed according to the WHO recommendations (67). We used the question “Do you have any CVD diagnosed by a doctor” to determine any diagnosed CVD, coded as “Any CVD.” We grouped individuals according to their educational background (1 = Primary; 2 = Secondary High school, incomplete higher education, or vocational school; and 3 = University degree). Individuals were grouped according to their BMI (weight in kilograms divided by the square of height in meters) as follows: BMI <25 kg/m^2^ as (1) reference; BMI ≥ 25 kg/m^2^ as (2) individuals with higher risk (68).

The Karolinska Sleep Questionnaire (KSQ) was used to assess sleep disturbance (69). The questions were: “Have you been bothered by the following complaints during the past three months”: “… difficulties falling asleep,” “… repeated awakenings with difficulties falling asleep again,” “… premature awakenings involuntary,” and “… disturbed/restless sleep.” The response options throughout the KSQ are (0) never, (1) seldom (occasionally), (2) sometimes (several times per month), (3) often, (4) most of the times, or (5) always. The score can range from 0 to 20 (high score representing high level of sleep disturbance). The KSQ has good reliability, construct validity, and criterion validity (69). The internal consistency in the current study was good (Cronbach's Alfa 0.868). For further analysis, the participants were divided into groups that as far as possible constituted the first to third and the fourth quartile: 82.7% individuals with score 0-8 (“less sleep problems”); and the rest with score 9 or higher (“much sleep problems”).

The physical and emotional subscale of the Shirom Melamed Burnout Questionnaire was used to measure burnout (70, 71). The subscale consists of eight items (“I feel tired,” “I feel refreshed,” “I feel physically exhausted,” “I feel fed-up,” “My batteries" are “dead", “I feel burned out,” “I feel mentally fatigued,” “I feel no energy for going to work in the morning”). The response scale ranges from 1—“almost never” to 7—“Almost always.” The score can thus range between 7 and 56 (high score representing high level of burnout). The SMBQ has good construct validity and reliability (72). The internal consistency in the current study was good (Cronbach's Alfa 0.848). For further analysis, we divided individuals into two groups according to quartiles: 76.5% individuals with score 8-25 as “low burnout”; and the remaining with score 26 or higher as “high burnout.”

The 10-item Perceived Stress Scale (PSS-10) was used to measure degree to which situations are appraised as stressful (73). The items assess how unpredictable, uncontrollable, and overloaded the respondents find their lives (“… been upset because of something that happened unexpectedly?” “…felt that you were unable to control the important things in your life?”, “…felt nervous and stressed?”, “…felt confident about your ability to handle your personal problems?”, “…felt that things were going your way?”, “…felt that you could not cope with all the things that you had to do?”, “…felt you been able to control irritations in your life?”, “…felt that you were on top of things?”, “.how often have you been angered because of things that happened that were outside of your control?”, “how often have you felt difficulties were piling up so high that you could not overcome them?”). The score can range from 0 to 40 (high score representing high stress level). The PSS-10 has good construct validity (74). The internal consistency in the current study was good (Cronbach's Alfa 0.901). For further analysis, we divided individuals into two groups according to quartiles: 75.6% individuals with score 0-21 (“low stress”); and the rest with score 22 or higher (“high stress”). Mean scores for all “mental distress factors” (sleep disturbance, burnout, perceived stress) were calculated (see Table 1).

**Table 1:** Prevalence of cardiovascular disease (CVD), socio-demographic outcomes, and mental distress factor in various ethnic groups.

	**Kyrgyz**	**East Europeans**	**Central Asians**	**East Asians**	**Western Asians**	**Other minorities**	**Chi^**2**^ (*p*)**
**CVD**, ***n*** **(%)**							17.29 (<0.001)
Yes	67 (30.9)	102 (42.7)	30 (26.3)	13 (34.2)	9 (39.1)	13 (20.6)	
No	150 (69.1)	137 (57.3)	84 (73.7)	25 (65.8)	14 (60.9)	50 (79.4)	
**Gender**, ***n*** **(%)**							24.46 (<0.001)
Male	76 (35.0)	97 (40.6)	54 (47.4)	21 (55.3)	12 (52.2)	42 (66.7)	
Female	141 (65.0)	142 (59.4)	60 (52.6)	17 (44.7)	11 (47.8)	21 (33.3)	
**Age**, ***n*** **(%)**							111.45 (<0.001)
18-29	66 (30.4)	19 (7.9)	42 (36.8)	12 (31.6)	4 (17.4)	30 (47.6)	
30-39	49 (22.6)	67 (28.0)	23 (20.2)	9 (23.7)	5 (21.7)	16 (25.4)	
40-49	37 (17.1)	46 (19.2)	22 (19.3)	2 (5.3)	6 (26.1)	7 (11.1)	
50-59	50 (23.0)	42 (17.6)	17 (14.9)	3 (7.9)	4 (17.4)	4 (6.3)	
≥60	15 (6.9)	65 (27.2)	10 (8.8)	12 (31.6)	4 (17.4)	6 (9.5)	
**Education**, ***n*** **(%)**							30.03 (<0.001)
Primary	10 (4.6)	14 (5.9)	14 (12.3)	0 (0.0)	2 (9.1)	7 (11.3)	
High school	103 (47.5)	114 (47.7)	67 (58.8)	24 (63.2)	14 (63.6)	39 (62.9)	
University degree	104 (47.9)	111 (46.4)	33 (28.9)	14 (36.8)	6 (27.3)	16 (25.8)	
**Income (soms)**, ***n*** **(%)**							20.56 (0.15)
≤ 8,000	29 (22.8)	75 (39.3)	18 (25.0)	7 (35.0)	5 (26.3)	10 (23.8)	
8,001-16,000	34 (26.8)	52 (27.2)	26 (36.1)	3 (15.0)	7 (36.8)	15 (35.7)	
16,001-30,000	35 (27.6)	37 (19.4)	18 (25.0)	6 (30.0)	3 (15.8)	11 (26.2)	
≥30,001	29 (22.8)	27 (14.1)	10 (13.9)	4 (20.0)	4 (21.1)	6 (14.3)	
**BMI**, ***n*** **(%)**							17.21 (0.00)
<25	124 (57.1)	99 (41.4)	61 (53.5)	25 (65.8)	11 (47.8)	37 (58.7)	
≥25	93 (42.9)	140 (58.6)	53 (46.5)	13 (34.2)	12 (52.2)	26 (41.3)	
**Mean score value (*****SD*****)**							***P***
Sleep disturbance	4.99 (4.02)	5.44 (4.12)	5.12 (3.75)	5.23 (4.05)	5.04 (3.39)	3.82 (3.73)	0.13
Burnout	19.77 (9.69)	20.19 (8.71)	19.29 (8.73)	17.97 (9.17)	20.52 (10.59)	17.36 (8.73)	0.27
Stress	16.44 (8.86)	16.33 (7.04)	16.36 (8.79)	14.73 (6.89)	15.95 (7.58)	12.93 (9.25)	0.05

### Statistical Analysis

The statistical analysis was performed using the IBM SPSS Statistics for Windows, Version 23.0. Armonk, NY: IBM Corp. Differences between ethnic groups in prevalence of CVD, socio-demographic outcomes and mental distress factor were tested with Chi-Square Test, *T*-Test, and *post-hoc* Bonferroni Test. Analyses of covariance (ANCOVAs) were conducted to study the associations between prevalence of CVD and levels of sleep disturbance, burnout, and stress. The odds for having CVD in relation to level of sleep disturbance, burnout, and stress in the various ethnic groups, with the Kyrgyz as referents, were assessed with logistic regression analysis, where also gender and BMI were considered. Confounding variables were only included in the analyses if they correlated with the analyzed variables according to Spearman correlation analysis. Since age was highly correlated with CVD (0.693, see Appendix 1), we removed age from further calculations. Due to multicollinearity, the independent variables with a high bivariate correlation should not be included in multiple regression analysis (75). The α-level was set at 0.05.

### Ethical Concerns

Ethical approval was received from the Research Ethics Committee of the International School of Medicine, Kyrgyzstan (Ref #10, 28.06. 2017). All study participants gave written informed consent in accordance with the Declaration of Helsinki.

## Results

There were significant differences in the distribution of CVD (χ^2^ = 17.29 at *DF* = 1, *p* < 0.001) and socio-demographic outcomes between the ethnic groups (Table 1). According to *post-hoc* tests, compared to Kyrgyz people (30.9%), the prevalence was significantly higher (*p* < 0.05) among East Europeans (42.7%), Western Asians (39.1%), and lower among Central Asians (26.3%) and other minorities (20.6%), in this study. Having high BMI (≥25) was more common among East Europeans and Western Asians. Among the mental distress factors, stress level showed a clear tendency to differ significantly (*p* = 0.05) between ethnic groups, whereas levels of sleep disturbance and burnout did not (Table 1). *Post-hoc* Bonferroni tests showed that East Asians and Other minorities had significantly lower prevalence of high stress than the Kyrgyz (*p* < 0.05). The results from the Spearman correlation analyses are given in Appendix 1, showing ethnic group, gender and BMI as confounding variables.

We explored the association between CVD and mental distress in all studied individuals. Income was excluded from the analyses since too many respondents had not answered that question. The ANCOVA on the associations between severity of mental distress and prevalence of CVD, showed a statistically significant difference in level of sleep disturbance between individuals with CVD (*M* = 6.50, *SD* = 3.96) and those without CVD (*M* = 4.36, *SD* = 3.81) [*F*_(47.43)_, *DF* = 1, *p* < 0.001]. The difference remained after controlling for ethnic group, gender and BMI (*F* for in between group = 23.25, *p* < 0.001). There was also statistically significant difference in burnout score between the CVD group (*M* = 21.48, *SD* = 9.43) and the reference group (*M* = 18.57, *SD* = 8.83) [*F*_(16.06)_, *DF* = 1, *p* < 0.001]. The difference remained after controlling for ethnic group, gender and BMI (*F* for in between group = 14.81, *p* < 0.001). The difference in stress score between the CVD group (*M* = 16.79, *SD* = 7.63) and the reference group (*M* = 15.55, *SD* = 8.46) did not show a trend [*F*_(3.54)_, *DF* = 1, *p* = 0.060]. The difference remained insignificant after controlling for ethnic group, gender, and BMI. Thus, in this study, sleep disturbance and burnout seem to be associated with CVD, but not stress.

The logistic regression analyses (Table 2) indicated that compared to individuals with low levels of sleep problems, individuals with high levels of sleep problems have 2.16 (95% CI 1.4–3.34) times higher odds of having a CVD. Compared to individuals with low levels of burnout, individuals with high levels of burnout have higher (1.58 95% CI 1.07–2.33) odds of having a CVD. There was no difference in chances of having a CVD among individuals with high level of perceived stress compared to low level of perceived stress group. As for the differences in ethnic groups, in analysis of burnout and stress, compared to the majority ethnic group Kyrgyz, East Europeans had 1.55 (95% CI 1.02–2.35—for burnout) and 1.52 (1.01–2.31 for stress) times higher odds of having a CVD. Furthermore, compared to Kyrgyz, Central Asians and Other minorities had a tendency for lower odds and Western Asians and East Asians had a tendency for higher odds of having a CVD in case of all mental distress factors. However, these associations need to be considered carefully considering the small number of representatives in some minority groups.

**Table 2:** Association between mental distress factors and cardiovascular disease in regression analysis, odds ratios (95% CI); models adjusted for ethnicity and gender, and BMI (*n* = 694; with CVD *n* = 234).

	**Sleep (much sleep problems *n* = 120)**	**Burnout (high burnout *n* = 165)**	**Perceived stress (high stress *n* =169)**
	OR (CI 95%)	OR (CI 95%)	OR (CI 95%)
**Mental distress factor**	2.16 (1.4–3.34)^***^	1.58 (1.07–2.33)^*^	1.14 (0.77–1.69)
**Ethnicity (ref Kyrgyz**, *n* = 217**)**
East European (239)	1.47 (0.97–2.24)	1.55 (1.02–2.35)^*^	1.52 (1.01–2.31)^*^
Central Asian (114)	0.88 (0.51–1.51)	0.80 (0.53–1.53)	0.88 (0.52–1.51)
East Asian (38)	1.59 (0.74–3.42)	1.69 (0.78–3.66)	1.59 (0.73–3.44)
Western Asia (23)	1.41 (0.53–3.73)	1.36 (0.53–3.52)	1.40 (0.54–3.60)
Other minorities (63)	0.70 (0.34–1.44)	0.70 (0.34–1.44)	0.70 (0.34–1.43)
**Gender** (female *n* = 392)	2.31 (1.61–3.30)^***^	2.32 (1.62–3.30)^***^	2.33 (1.63–3.34)^***^
**BMI** (BMI ≥ 25 *n* = 337)	1.14 (1.09–1.18)^***^	1.13 (1.09–1.18)^***^	1.14 (1.09–1.18)^***^

* *p < 0.05;*

*** *p < 0.001*.

## Discussion

This study addressed the differences in cardiovascular health as well as social and psychological determinants in Kyrgyzstan. As for the prevalence of CVD, the results confirm findings of earlier studies showing different CVD pattern among various ethnic groups (32, 37). Particularly, East-Europeans like Russians have been shown to have a higher prevalence of CVD compared to Central Asians (Kyrgyzs and Kazakhs) (33). This study indicated significantly higher prevalence of CVD among East Europeans (42.7%) and Western Asians (39.1%) compared to Kyrgyz people (30.9%). Furthermore, compared to these groups, the prevalence of CVD was significantly lower among Central Asians (26.3%) and Other minorities (Dungans and Uyghurs, 20.6%). This may be explained by the tendency of East Europeans and Western Asians, compared to Kyrgyzs and Central Asians (Kazakhs, Tajiks) and other minorities, showing lower CVD mortality attributable to dietary risk, including less alcohol consumption (12, 76, 77).

We also clarified the levels of mental distress factors among the ethnic groups in Kyrgyzstan. Based on studies of Salyers and Bond (78), Mezick et al. (61), Grandner et al. (62), and Slopen et al. (63), we expected ethnic differences in burnout and sleep disturbances. However, in the current study the levels of burnout or sleep disturbance did not differ among ethnic groups whereas such differences by ethnic groups have been shown in other contexts (79). *Vice versa*, in this study, the mean score value of stress was lower among East Asians (*M* = 14.7) and Other minorities (Dungans and Uygurs, *M* = 12.9) than among the Kyrgyzs, East Europeans, and Central Asians (*m* > 16.3). This suggests that the majority population, Kyrgyz, does not stand out as having particularly better mental health than the other ethnic groups. The conclusions on the ethnic differences in burnout and sleep disturbance differ from those from studies in the US (61, 79). Thus, the present finding that in Kyrgyzstan, compared to other ethnic groups, the majority of the population, Kyrgyzs do not stand out having better mental stress outcomes can be explained by the lower economic status of this group (80–82). Furthermore, the higher rate of mental stress in Kyrgyzs, East Europeans and Central Asians may be attributed to the fact that unlike these ethnic groups, East Asians (Koreans), Dungans and Uyghurs (Other minorities) have kept their religious practices (e.g., pray five times a day) throughout Soviet time till currently (83–85), and religious practices have been associated with positive mental health outcomes (86).

The ANCOVAs showed significantly higher levels of sleep disturbance and burnout in the CVD group compared to the referent group. This difference remained significant after controlling for ethnic group, gender and BMI. Our regression analysis confirmed that compared to the individuals with lower levels of sleep problems and burnout, individuals with high levels of either of these mental distress factors were more likely to have CVD. Earlier studies have shown that poor sleep can lead to disease (51, 52) and cause exhaustion disorder and stress (49). Thus, our findings of associations of CVD with sleep disturbance and burnout, but not with stress, are not surprising. Stress fluctuates strongly over time to cope with everyday hassles, which compromises the sensitivity of this variable. Despite of previous reports of declining of sleep disturbance with age (79, 87), our results did not find this negative correlation.

In our logistic regression analysis for burnout and stress we could see significantly higher odds of having a CVD among East Europeans compared to Kyrgyz; and a tendency for lower odds compared to Central Asians and Other minorities compared to Kyrgyz. Thus, next to the level of mental distress, gender and BMI, specificities in ethnic groups may be associated with the higher prevalence of CVD. The mechanisms behind the effect of ethnic group for mental distress factors and CVD needs further exploration. The cross-sectional design of this study does not enable to test of such cause and effect. Whereas, ethnic group can be expected to be a cause rather than effect in this context, the associations can well be bidirectional.

We believe that some of our results may be explained by insufficient number of minority participants. Despite stratified random sampling, ethnic groups differed significantly in age, gender, and education. The invitations for participation directed at patients visiting polyclinics and health care centers cannot be expected to have resulted in a fully representative sample of participants, which compromises the representativeness of the findings, in particular regarding the prevalence rates. Since results on prospective self-reported assessment of mental distress, such as sleep disturbance and burnout, lack in consistency (88), there is a need for further assessment as well as psychophysiological evaluation. We also asked the participants about having any CVD diagnosed by a doctor rather than using hospital records. This might have caused diagnosis bias, even if we specified CVD diagnosed by a doctor.

Nevertheless, the current study is one of the very few studies of health inequality among ethnic minorities in Central Asia. The current findings add value to the existing bulk of knowledge of mechanisms mediating the relationship between cultural, mental stress factors, and CVD. The present use of self-reported CVD and mental distress may in future research be complemented by hospital records of CVD diagnosis and psychophysiological measures related to distress.

## Conclusion

This study suggests that Kyrgyz people have lower prevalence of CVD, compared to East Europeans and Western Asians in Kyrgyzstan, and higher compared to Central Asians and Other minorities. In studied sample in Kyrgyzstan, individuals with relatively high level of sleep disturbance and burnout, more likely reported suffering from CVD. Next to the mental distress factors, gender and BMI, the characteristics of ethnic groups may be associated with the higher prevalence of CVD, as there were higher odds for CVD among East Europeans compared to Kyrgyz.

The high prevalence rates indicate the need of better diagnosis and treatment of CVD and burnout as well as improving sleep quality with public health measures, including stress management, restful environment, increased physical activity, and better nutrition. Based on the present and previous study outcomes, it can be concluded that there is a need for the development of a relevant approach in mitigating sleep disturbance and preventing burnout in the general population, not only in specific ethnic groups.

## Data Availability Statement

The datasets generated for this study are available on request to the corresponding author.

## Ethics Statement

Ethical approval was received from the Research Ethics Committee of the International School of Medicine, Kyrgyzstan (Ref #10, 28.06. 2017). All study participants gave written informed consent in accordance with the Declaration of Helsinki.

## Author Contributions

HA and KD contributed conception of the study and organized the data collection and analysis. KO and HO contributed design, methods of the study, and interpretation of the data. MN and SN contributed interpretation of the study findings. KO and HA performed the statistical analysis and wrote the first draft of the manuscript. HO and KD wrote sections of the manuscript. All authors contributed to the manuscript revision, read and approved the submitted version.

## Conflict of Interest

The authors declare that the research was conducted in the absence of any commercial or financial relationships that could be construed as a potential conflict of interest.

## Appendix

**Table A1:** Spearman correlation coefficients between cardiovascular disease and key analysis factors (*n* = 694).

**Any CVD**	**Gender**	**Age**	**Education**	**Family status**	**Ethnic group**	**Income**	**BMI**	**Sleep score**	**Burnout score**
Gender (0 = female, 1 = male)	0.202^***^									
Age	0.693^***^	0.266^***^								
Education	0.058	0.093^*^	0.077^*^							
Family status	0.007	0.13	0.180^***^	0.086^*^						
Ethnic group	–0.038	-0.172^**^	–0.046	–0.160^**^	0.018					
Income	–0.233	-.149^**^	–0.282^***^	0.124^**^	0.130^**^	–0.052				
BMI	0.334^***^	0.03	0.449^***^	0.021	0.119^**^	0.027	–0.006			
Sleep score	0.251^***^	0.119^**^	0.275^***^	0.072	–0.037	–0.032	–0.058	0.067		
Burnout score	0.154^***^	0.111^**^	0.175^***^	0.149^***^	0.063	–0.054	0.071	0.113^**^	0.469^***^	
Stress score	0.061	0.176^***^	0.071	0.103^***^	0.097^*^	–0.090^*^	0.096^*^	0.005	0.401^***^	0.524^***^

* *p < 0.05;*

** *p < 0.01;*

*** *p < 0.001*.
